# Comparison of Peri-Implant Clinicoradiographic Parameters among Non-Smokers and Individuals Using Electronic Nicotine Delivery Systems at 8 Years of Follow-up

**DOI:** 10.3290/j.ohpd.b2082123

**Published:** 2021-09-30

**Authors:** Saad Obaid Alazmi, Faris Jaser Almutairi, Bandar Awadh Alresheedi

**Affiliations:** a Assistant Professor, College of Dentistry, Qassim University, Qassim, Saudi Arabia. Performed the radiological examinations, administered the questionnaire to the participants, wrote, read and revised the manuscript.; b Assistant Professor, College of Dentistry, Qassim University, Qassim, Saudi Arabia. Performed the statistical analysis, compiled results, wrote and revised the manuscript.; c Assistant Professor, College of Dentistry, Qassim University, Qassim, Saudi Arabia. Performed the clinical and radiological examinations, compiled results, wrote, read and revised the manuscript.

**Keywords:** crestal bone loss, dental implant, electronic nicotine delivery systems, inflammation, probing depth, smoking

## Abstract

**Purpose:**

It is hypothesised the peri-implant soft-tissue inflammatory parameters (plaque index [PI], gingival index [GI], and probing depth [PD]) are poorer and crestal bone loss (CBL) higher around dental implants placed in electronic nicotine delivery systems (ENDS)-users than controls (individuals that had never consumed any form of tobacco). The aim of this study was to assess the peri-implant clinicoradiographic parameters among non-smokers and individuals using ENDS at 8 years of follow-up.

**Materials and Methods:**

Self-reported non-smokers and individuals habitually using ENDS were included. A questionnaire was used to gather information about age in years, sex (female or male), daily frequency and duration of use of ENDS, family history of smoking and/or vaping, daily toothbrushing and flossing and most recent visit to a dentist or dental hygienist. These patients were evaluated for peri-implant CBL, PD, PI, BOP. The mesial and distal CBL was measured on digital bitewing radiographs that were taken using the long-cone paralleling technique. Group comparisons were statistically assessed and the level of significance was set at p < 0.05.

**Results:**

One hundred twenty-seven individuals (92 males and 35 females) were included. Sixty-three individuals (46 males and 17 females) had used ENDS for 9.2 ± 0.8 years (group 1) and 64 (46 males and 18 females) did not use any nicotinic products (group 2). The mean ages of individuals in groups 1 and 2 were 34.2 ± 1.3 and 35.1 ± 0.5 years, respectively. In all patients, platform-switched dental implants with moderately rough surfaces were placed at bone level using an insertion torque of 30-–35 Ncm. In both groups, implants had a diameter of 4.1 mm and the lengths ranged between 11 and 14 mm. In groups 1 and 2, implants were in function for 8.8 ± 0.4 and 8.5 ± 0.2 years, respectively. There was no statistically significant difference in mPI, mBoP, PD, and mesial and distal CBL around implants in groups 1 and 2 at 8 years of follow-up.

**Conclusion:**

Dental implants can demonstrate stable clinicoradiographic status and can remain functionally stable in non-smokers and ENDS users, provided that strict home oral hygiene measures are adopted.

Oral rehabilitation of partially and completely edentulous individuals using dental implants is a reliable treatment strategy.^16^ Routine oral hygiene maintenance (OHM) is a fundamental requirement that plays a critical role in the clinicoradiographic stability of dental implants.^2^ In a 24-month follow-up study, Al-Amri et al^2^ investigated the effect of OHM on the peri-implant clinicoradiographic parameters in patients with dental implants. The 2-year follow-up results showed that dental implants can demonstrate a survival rate of 100%, provided oral hygiene is stringently maintained.^2^ Nevertheless, an often-criticised risk factor that has been associated with the etiopathogenesis of peri-implant diseases (peri-implant mucositis and peri-implantitis) is habitual use of nicotinic products. It is well known that habitual use of tobacco products (such as cigarette waterpipe smoking) enhances soft tissue inflammation and augments crestal bone loss, thereby predisposing vulnerable patients to peri-implant diseases (peri-implant mucositis and peri-implantitis).^1,3,5,9,10^ Individuals attempting to reduce or quit tobacco smoking often use alternate means to satisfy their desire to smoke tobacco.^6^ These include the use of nicotine chewing gum, patches, and nasal sprays.^9,11,16,24,30,41^

Use of electronic nicotine delivery systems (ENDS) is popular, particularly among adolescents and young adults.^9,16, 24,40^ Moreover, the general misconception that vaping is less injurious to overall health compared with conventional tobacco smoking induces individuals who had never used any form of tobacco to start using ENDS.^18,21,31,33^ However, studies^15,26,35^ have shown that vaping is not a safe substitute to smoking; the use of ENDS has been associated with medical complications such as respiratory, cardiovascular and hepatic diseases. From an oro-dental perspective, studies^12,23,32^ have shown that use of ENDS enhances the risk of periodontal diseases (such as periodontitis) in susceptible patient populations. Similarly, there is mounting evidence that habitual use of ENDS can augment soft tissue inflammation and crestal bone loss (CBL) around osseointegrated dental implants.^7,10,39^ Results from an experimental study^39^ showed that vapor from ENDS dysregulates the interaction between titanium implant surface and osteoblasts. This may interfere in the bone-to-implant contact, thereby compromising the long-term stability of dental implants. Moreover, use of ENDS has been associated with an increased growth and attachment of microbes, such as bacteria and yeasts, to gingival tissues.^4^ In a recent review, Alqahtani^8^ proposed that possibly increased oral colonisation of oral yeasts contributes to the etiopathogenesis of peri-implant diseases. In the present study, it is hypothesised that the peri-implant soft-tissue inflammatory parameters (plaque index [PI], gingival index [GI], and probing depth [PD]) are poorer and CBL is higher around dental implants placed in ENDS users compared with controls (individuals who have never consumed any form of tobacco).

The aim was to assess the peri-implant clinicoradiographic parameters among non-smokers and individuals using ENDS at 8 years of follow-up.

## Materials and Methods

### Ethical Approval and Consent to Participate

The present study was performed following guidelines recognised by the Declaration of Helsinki as revised in 2013 for experimentation involving human patients. Ethical approval was obtained from the ethics research committee of the Centre for Specialist Dental Practice and Clinical Research, Saudi Arabia (UDCRC/0047-2517). Participation was voluntary; there were no penalties and/or negative consequences associated with withdrawal from the present study. Signing the consent form was mandatory for all individuals.

### Inclusion and Exclusion Criteria

The inclusion criteria were as follows: a. non-smokers (individuals who reported to have never used any form of nicotinic product);^25,39^ b. individuals individuals who reported using ENDS such as electronic cigarettes (e.g. JUUL) at least once daily for the past 12 months);^6,15,38^ and c. signing the informed consent form. Former and current cigarette smokers, individuals using other forms of nicotinic products such as waterpipes (also known as narghile, hubble bubble and hookah), pipes, and cigars; individuals smoking nicotinic products and vaping; patients with self-reported systemic diseases such as diabetes mellitus (DM), cardiovascular diseases and renal/hepatic disorders; and individuals that had undergone antibiotic, probiotic, bisphosphonate and/or cancer therapy within the past 12 months were not included. Moreover, individuals that had consumed non-steroidal anti-inflammatory drugs within the past 6-months were excluded.

### Study Design and Dental Implants

The present study had a retrospective study design. According to the patients’ records, the dental implants assessed had been placed by private practitioners in the region.

### Participants and Grouping

Participants were divided into 2 groups: group 1: individuals habitually using ENDS; group 2: non-smokers.

### Questionnaire

A questionnaire was used to gather information about age in years, sex (female or male), daily frequency and duration of ENDS use, family history of smoking and/or vaping, daily toothbrushing and flossing, and most recent visit to a dentist or dental hygienist. Data pertaining to jaw location of implants, implant geometry, insertion torque, implant surface features (smooth or moderately rough), and duration of implants in function was retrieved from dental charts.

### Radiological and Clinical Parameters

The mesial and distal CBL was measured on digital bitewing radiographs that were taken using the long-cone paralleling technique.^19^ A positioner (X-ray Holders, KerrHawe; Bioggio, Switzerland) was positioned on the 30.5 x 40.5 mm film (Kodak-Ultraspeed size-II Dental-Film, Kodak; Rochester, NY, USA) parallel to the long axis of the implant and perpendicular to the x-ray cone.^19^ In summary, CBL was gauged as the linear distance from 2 mm under the abutment-implant interface up to the osseous crest.^3^ All radiographs were assessed by a calibrated investigator (Kappa score of 0.94). The soft tissue inflammatory parameters (PI, GI and PD) were assessed by one calibrated researcher (Kappa score 0.92). In groups 1 and 2, peri-implant PI and GI were measured using the protocol described by Löe.^37^ The PD was measured in millimeters using a graded plastic probe.

### Statistical Analysis

A software package was used to perform the statistical comparisons among the study groups (SPSS version 20; Chicago, IL, USA). Data normality was determined using the Shapiro-Wilk test. Group comparisons were performed using the Mann-Whitney U-test and Student’s t-test. p-values below 0.05 were considered statistically significant. The sample size was estimated based upon the results of a pilot investigation. It was estimated that inclusion of at least 30 individuals per group would be needed to give the study a power of 95% with an alpha error of 0.05.

## Results

### General Characteristics

One hundred twenty-seven individuals (92 males and 35 females) were included. Sixty-three individuals (46 males and 17 females) had used ENDS for 9.2 ± 0.8 years (group 1) and 64 (46 males and 18 females) did not use any nicotinic products (group 2). The mean ages of individuals in groups 1 and 2 were 34.2 ± 1.3 and 35.1 ± 0.5 years, respectively. In group 1, participants used electronic cigarettes for an average duration of 9.3 ± 0.5 years, and 41 (61.2%) individuals reported that they used ENDS because they thought vaping was less hazardous to overall health than smoking. In group 1, the participants used electronic-cigarettes 6.5 ± 0.4 times daily and each session involved 3.5 ± 0.2 puffs of vapor. Toothbrushing twice daily was reported by 82.5% and 85.9% in groups 1 and 2, respectively. Full-mouth interdental flossing twice daily was reported by 61.9% and 68.8% in groups 1 and 2, respectively. Biannual dental check-ups and prophylaxis was reported by 57.1% and 62.5% in groups 1 and 2, respectively (Table 1).

**Table 1:** Characteristics of the study cohort

Parameters	Group 1	Group 2
Patients (n)	63	64
Gender (M:F)	46:17	46:18
Age in years (mean ± SD)	34.2 ± 1.3 years	35.1 ± 0.5 years
Duration of ENDS usage	9.3 ± 0.5 years	NA
Reason for using ENDS		NA
They are less hazardous to health than cigarettes	41 (61.2%)	NA
They are fruity	22 (38.8%)	NA
No reason	—	NA
Daily frequency of vaping		
Number of times daily	6.5 ± 0.4 times/day	NA
Number of puffs per session	3.5 ± 0.2 puffs	NA
Daily tooth brushing		
Once daily	11 (17.5%)	9 (14.1%)
Twice daily	52 (82.5%)	11 (85.9%)
Flossing		
Once daily	24 (38.1%)	20 (31.2%)
Twice daily	39 (61.9%)	44 (68.8%)
Visits to oral healthcare provider		
Annually	23 (36.5%)	18 (28.1%)
Biannually	36 (57.1%)	40 (62.5%)
When needed	4 (6.4%)	6 (9.4%)

Group 1: Patients habitually using ENDS; group 2: Patients not using any nicotinic products; M: male; F: female; *All implants were placed in the regions of missing premolars or molars.

### Characteristics of Implants

Seventy-five and 82 implants were placed in groups 1 and 2, respectively. In group 1, 35 implants were placed in the mandible and 40 in the maxilla. Forty and 42 implants were placed in the mandible and maxilla, respectively, in group 2. In groups 1 and 2, implants had been in place for 8.8 ± 0.4 and 8.5 ± 0.2 years, respectively. All implants were platform-switched with moderately rough surfaces and had been placed at bone level using an insertion torque of 30–35 Ncm. Implants had a diameter of 4.1 mm and the lengths ranged between 11 and 14 mm (Table 2).

**Table 2:** Peri-implant clinicoradiographic parameters in the study group

Parameters	Group 1	Group 2
Number of implants	75	82
Jaw location (mandible:maxilla)	35:40	40:42
Duration of implants in function	8.8 ± 0.4 years	8.5 ± 0.2 years
Plaque index	1.05 ± 0.08	1.2 ± 0.05
Gingival index	0.8 ± 0.06	0.7 ± 0.04
Probing depth	1.8 ± 0.1 mm	1.6 ± 0.03 mm
Crestal bone resorption (mesial)	1.4 ± 0.04 mm	1.05 ± 0.06 mm
Crestal bone resorption (distal)	1.3 ± 0.05 mm	1.2 ± 0.07 mm
Implant loss	None	None

### Peri-implant Parameters at 8-year Follow-up

There was no statistically significant difference in peri-implant PI, GI, PD and mesial and distal CBL in groups 1 and 2 at 8 years of follow-up. None of the implants were lost up to 8 years of follow-up (Table 2). There was no statistically significant difference in peri-implant PI, GI, PD and mesial and distal CBL around implants placed in the maxilla and mandible in groups 1 and 2 at 8 years of follow-up (Table 3).

**Table 3:** Peri-implant parameters in relation to jaw location

Peri-implant parameters	Group 1		Group 2	
	Maxilla	Mandible	Maxilla	Mandible
Number of implants	40	35	42	40
Plaque index	1.2 ± 0.04	1 ± 0.007	1.4 ± 0.007	0.9 ± 0.06
Gingival index	0.7 ± 0.06	0.9 ± 0.04	0.6 ± 0.05	1.04 ± 0.004
Probing depth	2.05 ± 0.1 mm	1.6 ± 0.04 mm	1.5± 0.04 mm	1.9 ± 0.1 mm
Crestal bone resorption (mesial)	1.7 ± 0.07 mm	1.3 ± 0.1 mm	1.4 ± 0.08 mm	0.9 ± 0.005 mm
Crestal bone resorption (distal)	1.5 ± 0.05 mm	1.1 ± 0.06 mm	1.5 ± 0.07 mm	1 ± 0.004 mm
Implant loss	None	None	None	None

## Discussion

There is abundant evidence from experimental studies which confirm that habitual use of nicotinic products delays the cellular healing response.^20,35^ In a systematic review and meta-analysis of pre-clinical studies, Ghanem et al^20^ investigated the impact of nicotine on the osseointegration of implants. Results from over 60% of the studies assessed in this systematic review and meta-analysis showed no statistically significant effect of nicotine on healing around implants.^20^ However, quantitative assessment showed that nicotine compromises bone-to-implant contact.^20^ Since vapor from ENDS has been reported to dysregulate the adhesion of osteoblasts to titanium implant surfaces, along with the fact that nicotine compromises the morphology and function of human gingival fibroblasts,^39^ the authors of the present study hypothesised that peri-implant soft-tissue inflammatory parameters are poorer and CBL is higher around implants placed in ENDS users compared with controls. Interestingly, results of the present study led to rejecting the hypothesis, as no statistically significant difference in PI, GI, PD and mesial and distal CBL was observed around implants placed in both ENDS users (group 1) and non-smokers (group 2). It may therefore be speculated that that nicotine does not compromise the stability of dental implants. In a systematic review, Holliday et al^21^ concluded that the amounts of nicotine to which ENDS users are exposed to is less likely to have toxic effects on human periodontal and gingival cells. Likewise, in an experimental study on rabbits, Linden et al^36^ showed that subcutaneous injection of nicotine after implant insertion did not affect osseointegration. Similar results have been reported in other experimental studies.^14,42^ This seems to offer an explanation for the comparable clinicoradiographic parameters that were observed among patients in groups 1 and 2. However, it is important to interpret these results with extreme caution, as by no means do the authors intend to claim that vaping is a safe alternative to conventional tobacco smoking or that vaping does not compromise the integrity of oral soft and osseous tissues.

A number of factors may have contributed to the results of the present study. Firstly, it is pertinent to note that oral hygiene maintenance was routinely performed by all participants in groups 1 and 2. In the present study, at least 82% in groups 1 and 2 reported that brushing twice daily, and at least 60% in both groups used dental floss around teeth and implants twice a day. This suggests adequate plaque control around teeth and dental implants. This factor may have contributed to reducing the risk of periodontal and peri-implant diseases in the population under assessment. It is also possibley that after having lost their natural teeth and having them replaced by dental implants, the patients more vigilantly maintained routine oral hygiene measures. Moreover, it is plausible that nearly 57% and 62% in groups 1 and 2 visited their oral healthcare providers biannually (most likely every 6 months), probably for routine check-ups and prophylaxis. It is speculated that during routine dental visits, these individuals underwent full-mouth plaque and/or calculus removal using traditional prophylactic methods, such as ultrasonic scaling. This suggests that the daily oral hygiene maintenance protocols adopted by the patients and professional evaluation and prophylaxis by oral healthcare providers played a role in the clinicoradiographic stability of dental implants placed in ENDS users and controls alike. The authors support the results from previous studies,^5,39^ in which no statistically significant differences in periodontal inflammatory parameters were observed among non-smokers and ENDS users compared with cigarette smokers. This suggests that peri-implant clinicoradiographic parameters may also remain stable among ENDS users, provided stringent OHM measures are kept. The literature contains abundant evidence that high educational status is directly associated with a superior oral health status.^27^ It is therefore speculated that all participants included in the present investigation were educated enough to comprehend the significance of oral hygiene maintenance, which leads to the long-term survival of dental implants without complications. This also suggests that patient education and routine dental follow-ups/prophylaxis are critical for maintaining healthy periodontal and peri-implant soft tissue and crestal bone levels. The present authors concur with the study by Tran et al,^43^ the authors of which proposed that a lack of professional maintenance is significantly associated with implant failure.

There are a number of limitations related to the present study. Firstly, the authors relied upon self-reported data regarding the use of ENDS. In this context, it is imperative to chemically validate the nicotine-intake status (smoking or ENDS usage) by assessing biomarkers such as salivary cotinine levels.^38^ Such chemical validation may help precisely define patient groups such as non-smokers and ENDS users. Moreover, stringent eligibility criteria were imposed in the present study in relation to the selection of study participants. Tobacco smokers and immunosuppressed individuals were excluded. Likewise, a state of persistent hyperglycemia, which is a common manifestation among patients with poorly controlled DM, is also a risk factor for periodontal and peri-implant diseases.^ 11,27,29^ Moreover, smoking and impaired glycemic levels are also risk factors of early graft failure in susceptible patients.^35,43^ Further studies are needed to assess the influence of glycemic control and quitting tobacco smoking on the survival of dental implants in ENDS users and non-smokers.

## Conclusion

Dental implants can demonstrate a stable clinicoradiographic status and remain functionally stable in non-smokers and ENDS users, provided strict domestic oral hygiene measures are kept and routine dental prophylaxis is carried out by oral healthcare providers.
